# Neferine Ameliorates Slow-Transmitting Constipation by Inducing PINK1/Parkin-Mediated Mitophagy in Protective Enteric Glial Cells

**DOI:** 10.4014/jmb.2412.12022

**Published:** 2025-04-27

**Authors:** Taiyu Chen, Xiaodong Jiang, Bo Ma, Yu Zhan, Yong Wen, Lifang Mao, Jun Pang, Xuegui Tang

**Affiliations:** 1 Anorectal Department of Integrated Traditional Chinese and Western Medicine, Affiliated Hospital of North Sichuan Medical College, Nanchong 637100, Sichuan, P.R. China; 2 Anorectal Department, Langzhong People's Hospital, Langzhong 637400, Sichuan, P.R. China; 3 Anorectal Department, Chengdu First People’s Hospital, Chengdu 610041, Sichuan, P.R. China; 4 Affiliated Hospital of Integrative Chinese Medicine and Western Medicine of Chengdu University of TCM, Chengdu 610075, Sichuan, P.R. China; 5 Department of Traditional Chinese Medicine, The Affiliated Hospital of Southwest Medical University, Luzhou 646000, Sichuan, P.R. China; 6 North Sichuan Medical College, Integrated Traditional Chinese and Western Medicine College, Nanchong 637100, Sichuan, P.R. China; 7 Anorectal Department, Affiliated Hospital of Chengdu University of Traditional Chinese Medicine, Chengdu 610075, Sichuan, P.R. China

**Keywords:** Neferine, enteric glial cells, slow-transmitting constipation, mitophagy, PINK1/Parkin signaling pathway

## Abstract

The enteric glial cells (EGCs) are the main components of the enteric nervous system (ENS) and contribute to the development of slow transit constipation (STC). In this study, we aimed to explore the effects of neferine (Nef) on EGCs based on PINK1/Parkin-mediated mitophagy. In vivo, 7 days of loperamide feeding was conducted to model STC rats, which were then treated with 2.5, 5, 10 mg/kg/d Nef, and 2 mg/kg/d mosapride for 14 days. In vitro, a CCK-8 assay was performed to detect EGC viability. EGCs were then stimulated by 400 μM H_2_O_2_, transfected with si-PINK1, and treated with Nef or mitochondrial division inhibitor 1 (Mdivi-1). Colon tissue was observed by H&E staining, TEM, ELISA (to quantify SOD, MDA, GDNF, and NGF expression), and immunofluorescence (to count the number of mitochondria). In addition, flow cytometry was used to quantify cell apoptosis, ROS, and mitochondrial membrane potential (MMP). Finally, the p62, PINK1, Parkin, and LC3II/I expression levels were measured by western blotting. Nef was shown to significantly improve STC in rats and reduce mucosal epithelial cell loss, inflammatory cell infiltration, and fibrous proliferation. Moreover, Nef reduced ROS and MDA levels while increasing SOD, GDNF, and NGF. Nef treatment also increased the LC3II/I ratio, as well as p62, PINK1, and Parkin expression, which helped mitigate mitochondrial expansion. However, PINK1 silencing shared the same function as Mdivi-1 in the STC+Nef group, inhibiting EGC viability, oxidative stress, and PINK1/Parkin signaling activation. Additionally, mitophagy was exacerbated by si-PINK1 in the STC+Nef group EGCs. In short, Nef ameliorates STC by inducing PINK1/Parkin-mediated mitophagy in EGCs.

## Introduction

Slow transit constipation (STC) refers to refractory constipation caused by weakened colonic motility, leading to the slow transit of contents through the colon [1]. Currently, constipation has a relatively high global prevalence, affecting approximately 10.3%-45.5% of the population, with about half of these cases being STC [2, 3]. As living standards improve, the incidence of STC has shown an upward trend. It is well known that lifestyle adjustments form the foundation of treatment for STC, but for severe cases, medication intervention is still necessary. Existing drugs, such as prucalopride, tegaserod, and stimulant laxative bisacodyl, are associated with serious side effects [4]. The 5-HT4 receptor agonist mosapride is commonly used in clinical practice to treat STC, and offers the advantage of fewer side effects [5]. However, the efficacy varies from person to person, and it is often less effective in patients with severe constipation. Therefore, finding alternative medications to mosapride is crucial for clinical STC treatment.

The enteric nervous system (ENS) is a network-like structure distributed throughout the gastrointestinal wall. Modern pathological research has discovered that abnormalities in the ENS are the primary pathological manifestation in patients with STC, and enteric glial cells (EGCs) are the main components of the ENS [6, 7]. As reported, EGCs play a neurotrophic role by wrapping enteric neurons to protect the neuronal network to maintain normal peristalsis [8, 9]. Thus, EGC loss is seen as the key factor in gastrointestinal motility problems. Previous research suggested that autophagy may be involved in the process of EGC loss during STC occurrence [10]. Mitophagy is a crucial form of autophagy regulated by the PTEN-induced putative kinase 1 (PINK1)/Parkin signaling pathway. We hypothesize that activating mitophagy in EGCs could be a potential therapeutic target for treating STC.

Neferine (Nef) is a major benzylisoquinoline alkaloid derived from the green embryo of lotus seeds. It exhibits strong antioxidant and neuroprotective effects with potential use in treating depression, epilepsy, Alzheimer’s disease, and ischemic brain injury [11-13]. Besides their gastrointestinal regulatory properties, lotus seeds are also reported to have astringent and antidiarrheal properties. Nef has also been shown to treat ulcerative colitis [14]. Moreover, there are reports that lotus seed oligosaccharides can increase the concentration of short-chain fatty acids in the gut, thus alleviating constipation in mice [15]. Nevertheless, the regulation of gastrointestinal function by lotus seeds may be bidirectional, and the effect of Nef on STC remains unclear. Considering the pharmacological effects, Nef has been inferred to be a potential drug for STC treatment. However, as the underlying macular mechanism remains unclear, we therefore investigated whether Nef can protect EGCs from STC damage based on mitophagy and the induction of the PINK1/Parkin signaling pathway.

## Materials and Methods

### STC Animal Modeling and Drug Treatment

In total, 36 male SD rats weighing 180-220 g, aged 56 days, were purchased from the Chengdu Dashuo Co.(SCXK (chuan)2020-0030). After acclimatization feeding for 7 days, rats were randomly divided into groups (*n* = 6): control, STC, STC+L-Nef, STC+M-Nef, STC+H-Nef, and STC+mosapride. For 7 consecutive days, 15 mg/kg/day loperamide (Lop) was used to feed STC rats via oral gavage. Next, drug treatment groups were given 2.5 mg/kg/d, 5 mg/kg/d, 10 mg/kg/d Nef, and 2 mg/kg/d mosapride via oral gavage for 14 days, respectively. Finally, the amount of defecation and fecal water content (%) were recorded. Intestinal propulsion rate (%) was used to measure gastrointestinal transit. These indices were used in this study to examine the degree of STC.

### Charcoal Meal Test

Using a charcoal meal test, the gastrointestinal transit was evaluated. Rats were deprived of food for 18 h at the conclusion of the experimental day. After 10 min, they were given a meal containing activated charcoal (10%active charcoal and 5% carboxymethyl cellulose). After 30 min, the stomach and intestine were co-harvested from euthanized rats to assess the transit distance of the activated charcoal.

### STC Cell Modeling and Drug Treatment

EGCs (CL0380, Fenghbio, China) were cultured by high glucose DMEM medium (PM150210, Procell, China) at 37°C and 5% CO_2_. Then, 360 μl of 1X riboFECT CP Buffer was added to dilute 15 μl of siRNA. Following that, 36 μl of riboFECT CP Reagent was added to create a 50 nM riboFECT CP solution, which was then incubated at room temperature for 15 min. After trypsin digestion, EGC cells were collected in the logarithmic growth phase and washed with PBS. The cell density was then adjusted to 1 × 10^5^ cells/ml, and 2 ml/well was seeded in a 6-well plate. Next, the cells were cultured until they adhered to the surface. Then, riboFECT CP was added to transfect EGCs to silence PINK1.

The EGCs were induced by 400 μM H_2_O_2_ to model STC *in vitro* for 2 h. Treatment groups were treated with 10 μM Nef, 25 μM Mdivi-1(mitophagy inhibitor), and transfected with si-NC and si-PINK1, respectively. Groups: control, STC, STC+Nef, STC+Nef+si-NC, STC+Nef+si-PINK1, and STC+Nef+Mdivi-1.

### Hematoxylin and Eosin (H&E) Staining

The colon was fixed in 10% neutral formaldehyde for over 24 h, dehydrated using an ascending alcohol series, cleared twice in xylene for 5 min each, and embedded in paraffin. Then, samples were sliced into 5 mm sections and stained with hematoxylin and eosin (Solarbio, China). The results of the staining were visualized under a light microscope.

### Cell Counting Kit-8 (CCK-8)

For this assay, 2×10^5^ cells/ml EGCs were seeded in a 96-well plate and cultured until they adhered to the surface. Drug treatments are listed as follows: Experiment 1: 0, 5, 10, 15, and 20 μM Nef-treated EGCs for 12 h. Experiment 2: control, STC, STC+Nef, STC+Nef+si-NC, STC+Nef+si-PINK1, STC+Nef+Mdivi-1. A CCK-8 kit (BS350B, Biosharp, China) was used to detect the cell viability of EGCs. The absorbance at 450 nm was measured using a multifunctional microplate reader (ELx800, BioTek, USA).

### Molecular Docking

To analyze binding affinities between Nef and PINK1, the Nef molecular structure was downloaded from PubChem Compound (https://pubchem.ncbi.nlm.nih.gov/), and the 3D coordinates of PINK1 were downloaded from PDB (http://www.rcsb.org/pdb/home/home.do). AutodockVina 1.2.2 (http://autodock.scripps.edu/) was used to perform molecular docking.

### Enzyme-Linked Immunosorbent Assay (ELISA)

The Rat Glial Cell Line-Derived Neurotrophic Factor (GDNF) ELISA Kit (ZC-36814, ZCBIO, China), the Rat Nerve Growth Factor (NGF) ELISA Kit (ZC-37163, ZCBIO, China), the Superoxide Dismutase (SOD) ELISA Kit (A045-2-1, Nanjingjiancheng, China), and Malondialdehyde (MDA) (A003-1, Nanjingjiancheng, China) were employed in this study, to measure the GDNF, NGF, SOD, and MDA concentrations in the colon, and the GDNF and NGF concentrations of EGCs in each group. The ELISA procedures were performed according to the manufacturers’ protocols. The absorbance at 450 nm was measured using a SpectraMAX Plus384 multifunctional microplate reader (Molecular Devices, USA). Each experiment was conducted three times at least.

### Immunofluorescence (IF) Confocal Detection

MitoTracker Red (C1046, Beyotime, China) was used to incubate the EGC climbing slices for 30 min at 37°C. After the cells were washed twice with PBS, Lyso-Tracker Red was used to stain the slices for 50 min at 37°C. The colon slices were incubated with DHE for 30 min in the dark. For double IF, the colon slices were incubated with the first primary antibody against glial fibrillary acidic protein (GFAP) (1:100, GB11096, Servicebio, China) overnight at 4°C, followed by the second antibody (1:100, GB23303, Servicebio, China) and 30 min incubation. Then, the colon slices were added with FITC-Tyramide (1:500, G1222, Servicebio, China) and incubated for 10 min. After three washes with PBS, sections were incubated with the second primary antibody against Microtubule-associated protein 1A/1B-light chain 3 (LC3) (1:200, 14600-1-AP, Proteintech, USA), Parkin (1:200, 14060-1-AP, Proteintech, USA), and PINK (1:500, 23274-1-AP, Proteintech). DAPI (G1012, Servicebio, China) was used to stain the nucleus. The A1 confocal microscope (Nikon, Germany) was used to scan EGC climbing slices or colon slices.

### Transmission Electron Microscopy (TEM)

TEM was used to evaluate mitochondrial injury of the colon under the STC model and drug treatment. Samples were collected, fixed, and dehydrated. After the embedding and polymerization in Epon-812 resin, samples were cut into 70 nm. The sections were then stained with uranium acetate (GS02624, Beijing Zoomonkeyi Technology Co., Ltd., China) for 15 min, followed by staining with lead citrate (GA10701-1, Beijing Zoomonkeyi Technology Co., Ltd.) for 2 min. Image acquisition was performed using a JEM-1400FLASH TEM (Jeol, Japan).

### Flow Cytometry (FCM) Detection of Cell Apoptosis, ROS, and Mitochondrial Membrane Potential (MMP)

EGCs were collected after drug treatment or si-RNA transfection to detect apoptosis, ROS, and MMP by FCM. For cell apoptosis detection (KGA1030, Keygenshop, China), the cell sediments were resuspended with 500 μl binding buffer and stained by 5 μl Annexin V and 5 μl PI, respectively. The mix was incubated for 15 min in the dark at 25°C. For ROS detection (S0033S, Beyotime, China), the cell sediments were resuspended with 10 μmol/l DCFH-DA and incubated for 20 min at 37°C. The positive control group was stained with Rosup for 30 min at 37°C. Then, the cells were washed with medium (without serum) and resuspended with PBS. Finally, for MMP detection (C2006, Beyotime, China), 2 μl JC-1 was dissolved in 900 μl deionized water and 100 μl 10× incubation buffer as 1 ml JC-1 work solution. Cells were stained by JC-1 work solution and incubated for 20 min at 37°C. The incubation buffer was used to resuspend cells after two washes. A flow analyzer (Cytoflex, Beckman, USA) was used to conduct FCM.

### Western Blot

EGCs were collected in EP tubes and colon samples were ground. The RIPA solution was used to lysate cells and tissue for 30 min at 4°C. The protein concentration was determined using a BCA kit (P0009, Beyotime, China). Then, 20 μg protein was separated by SDS-PAGE electrophoresis and delivered to an immobilon-PSQ PDAF membrane (ISEQ00010, Sigma-Aldrich, USA). Membranes were incubated with primary antibodies against LC3 (1:1000, A7198, ABclonal, China), Parkin (1:1000, A0968, ABclonal), PINK1 (1:2000, BS-22173R, Bioss, China), p62 (1:2000, AF5384, Affinity, China), and β-actin (1:50000, AC026, ABclonal) overnight at 4°C. After 3 washes with PBS, membranes were incubated for 30 min with Goat Anti-Rabbit IgG (H+L) HRP (1:5000, S0001, Affbiotech, China) at room temperature. As a result, the target bands were detected using enhanced chemiluminescence (ECL).

### Statistical Analysis

Statistical analysis and graphing were performed using Prism 8.0 software. Data were expressed as mean ± SD. One-way ANOVA was used to compare means among multiple samples. LSD was applied if variances were homogeneous; Tamhanés T2 test was used if variances were heterogeneous. A *p*-value of <0.05 was considered to indicate a statistically significant difference between groups.

## Results

### Nef Improves Loperamide Hydrochloride (lop)-Induced STC in SD Rats

**Fig. 1:**
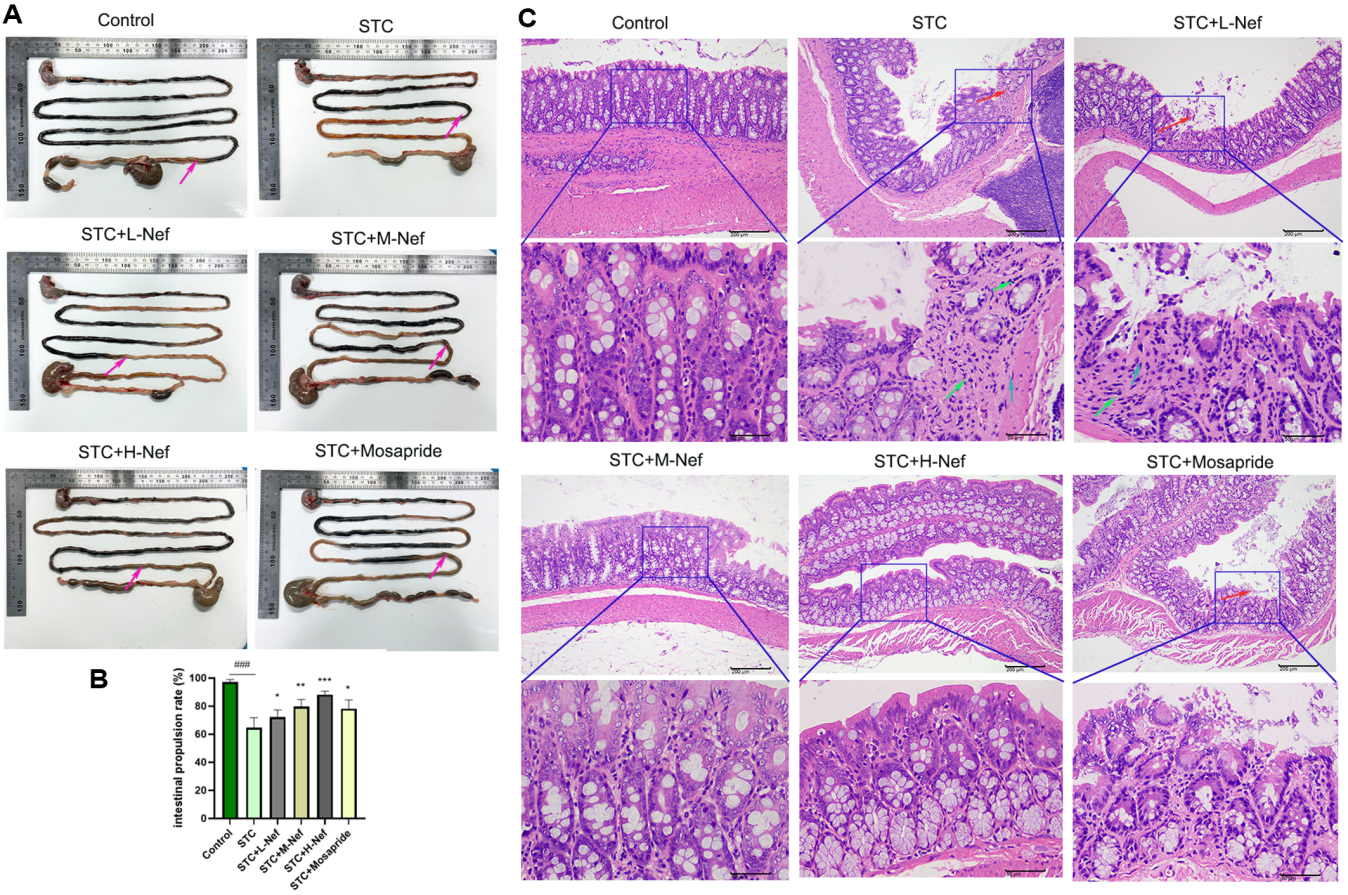
Nef improves loperamide hydrochloride (lop)-induced STC. (**A**) Charcoal meal test. (**B**) Fecal water content (%). (**C**) HE staining for the colon of each group (100×, 400×). Compared with the control group, ###*p* < 0.001; Compared with the STC group, *p < 0.05, **p < 0.01, ***p < 0.001.

The body weight, fecal excretion, and fecal water content (%) of rats are displayed in Table 1. There was no significant difference in the body weight of the rats in each group before the experiment was performed. After STC modeling, all indices were down. However, the body weight, fecal excretion, and fecal water content (%) increased by L-, M-, and H- doses of Nef or mosapride compared to the STC group. In this study, we found that Nef has a favorable therapeutic effect on STC. A charcoal meal test was used to measure the intestinal propulsion rate of the STC rats (%) (Fig. 1A). Fig. 1B showed that the intestinal propulsion rate (%) was decreased by Lop but increased by Nef or mosapride. It is necessary to observe the pathological phenomenon of the colon in each group to determine the effects of Nef on STC rats. As shown in Fig. 1C, compared with the control group, the STC group expressed a serious mucosal epithelial cell loss (red arrows), a small amount of inflammatory cell infiltration (green arrows), and fibrous tissue proliferation (blue arrows). However, the M-, and H- doses of Nef and mosapride treatment alleviated all mucosal epithelial cell loss, inflammatory cell infiltration, and fibrous tissue proliferation. These results suggest that Nef can normalize the function and structure of the colon in STC rats.

### Nef Inhibits Oxidative Stress of the Colon in Lop-Induced STC Rats

**Fig. 2:**
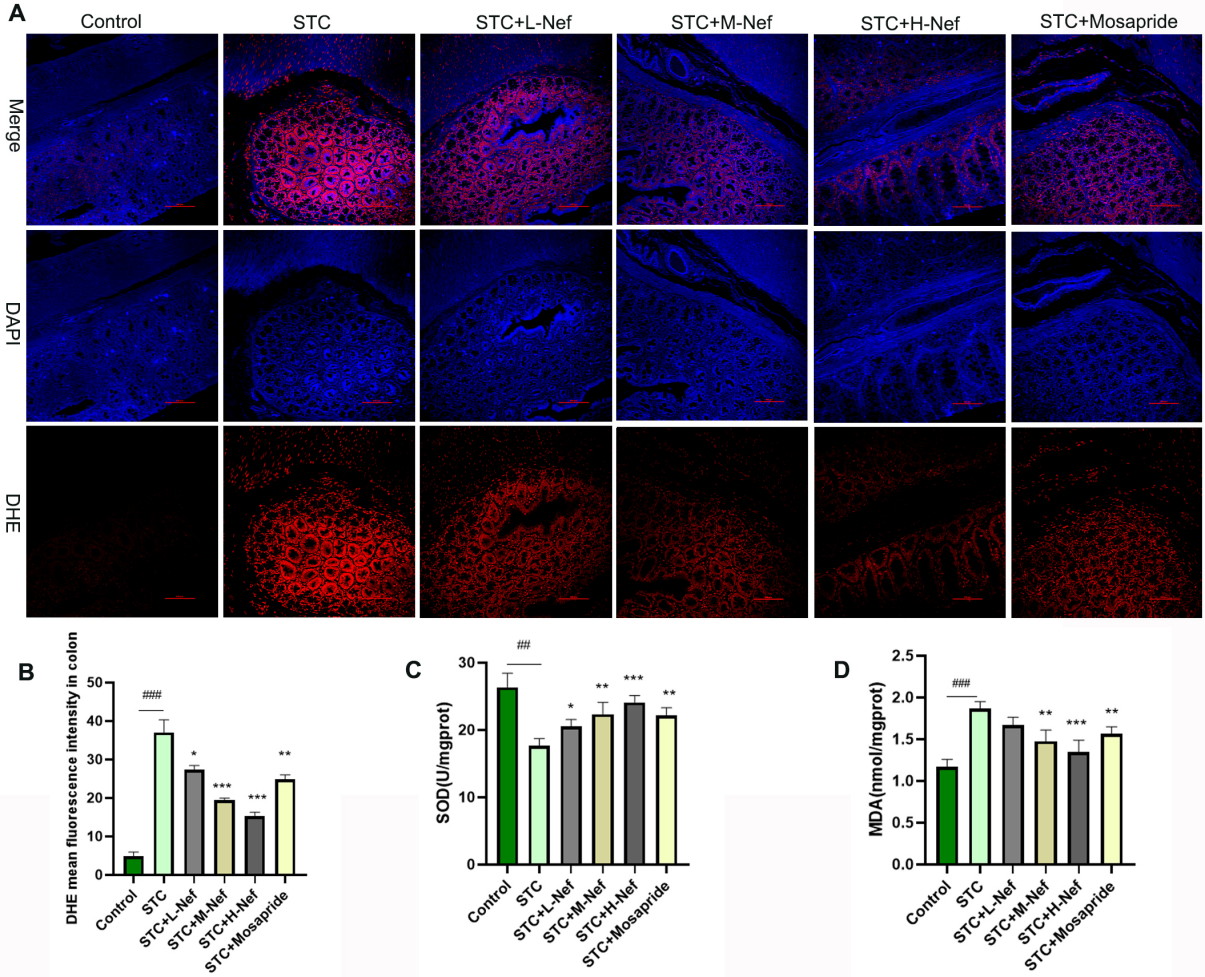
Nef inhibits oxidative stress in the colon in STC rats. (**A-B**) Immunofluorescence confocal assay for DHE to assess ROS levels. ELISA was performed to detect SOD (**C**) and MDA (**D**) levels in the colon. Compared with the control group, ##*p* < 0.01, ###*p* < 0.001; Compared with the STC group, *p < 0.05, **p < 0.01, ***p < 0.001.

ROS, SOD, and MDA are classical indices of oxidative stress. In this study, we performed a DHE immunofluorescence confocal assay to detect ROS levels in the colon tissue (Fig. 2A), while the red fluorescence detected ROS. Results showed that ROS (Fig. 2B) and MDA (Fig. 2D) expressions were upregulated, and SOD (Fig. 2C) expression was downregulated in the STC group in comparison to the control group. However, when compared with the STC group, different doses of Nef and mosapride treatment all caused a decrease in ROS and MDA, and an increase in SOD. Interestingly, the effects of Nef were dose-dependent. This suggests that Nef can block oxidative stress in the injured colon.

### Nef Activates the Pink1/Parkin-Mediated Mitophagy of EGCs in STC Rats

**Fig. 3:**
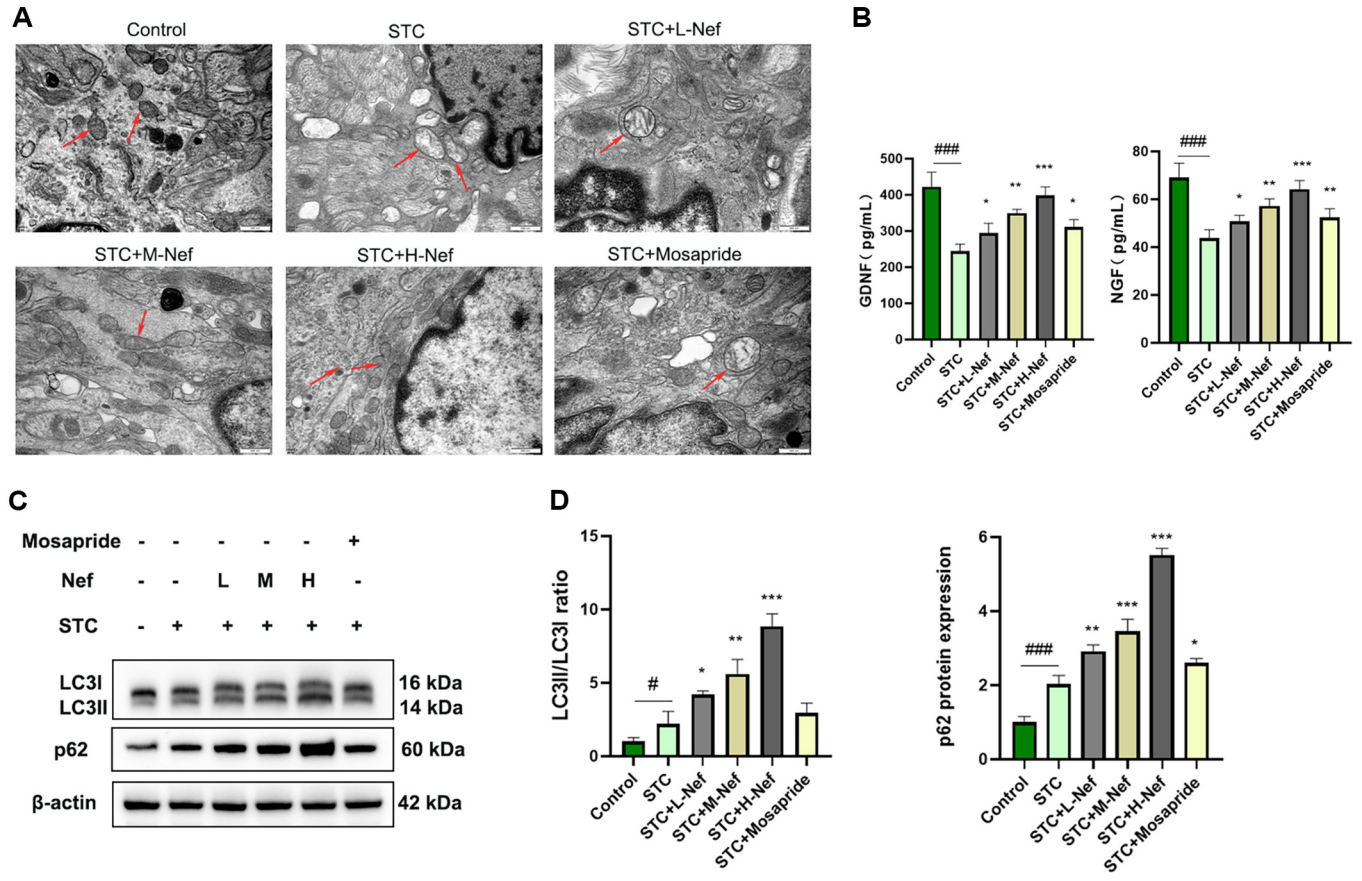
Effect of Nef on mitochondrial autophagy in EGCs in STC rats. (**A**) TEM was used to observe mitochondria in EGCs, bar = 500 nm. (**B**) ELISA was performed to detect GDNF and NGF levels. (**C-D**) p62 and LC3 proteins were quantified by WB. Compared with the control group, #*p* < 0.05, ###*p* < 0.001; Compared with the STC group, *p < 0.05, **p < 0.01, ***p < 0.001.

TEM was conducted to observe mitochondria in EGCs (red arrow). As shown in Fig. 3A, the STC and STC+L-Nef groups expressed serious mitochondrial swelling, and the mitochondria in the other group were normal. ELISA was used to detect GDNF and NGF levels in the colon of each group (Fig. 3B). Results revealed Lop-induced downregulation of GDNF and NGF expression, and Nef reversed it dose-dependently, as did mosapride. Since EGCs are tightly linked to STC, it is necessary to investigate whether Nef protects EGCs in STC rats. p62 and LC3 proteins were quantified by WB (Fig. 3C). Fig. 3D shows that lop increased the LC3II/LC3I ratio and p62 protein expression. However, compared with the STC group, the L, M, and H doses of Nef have a higher level of above all.

**Fig. 4:**
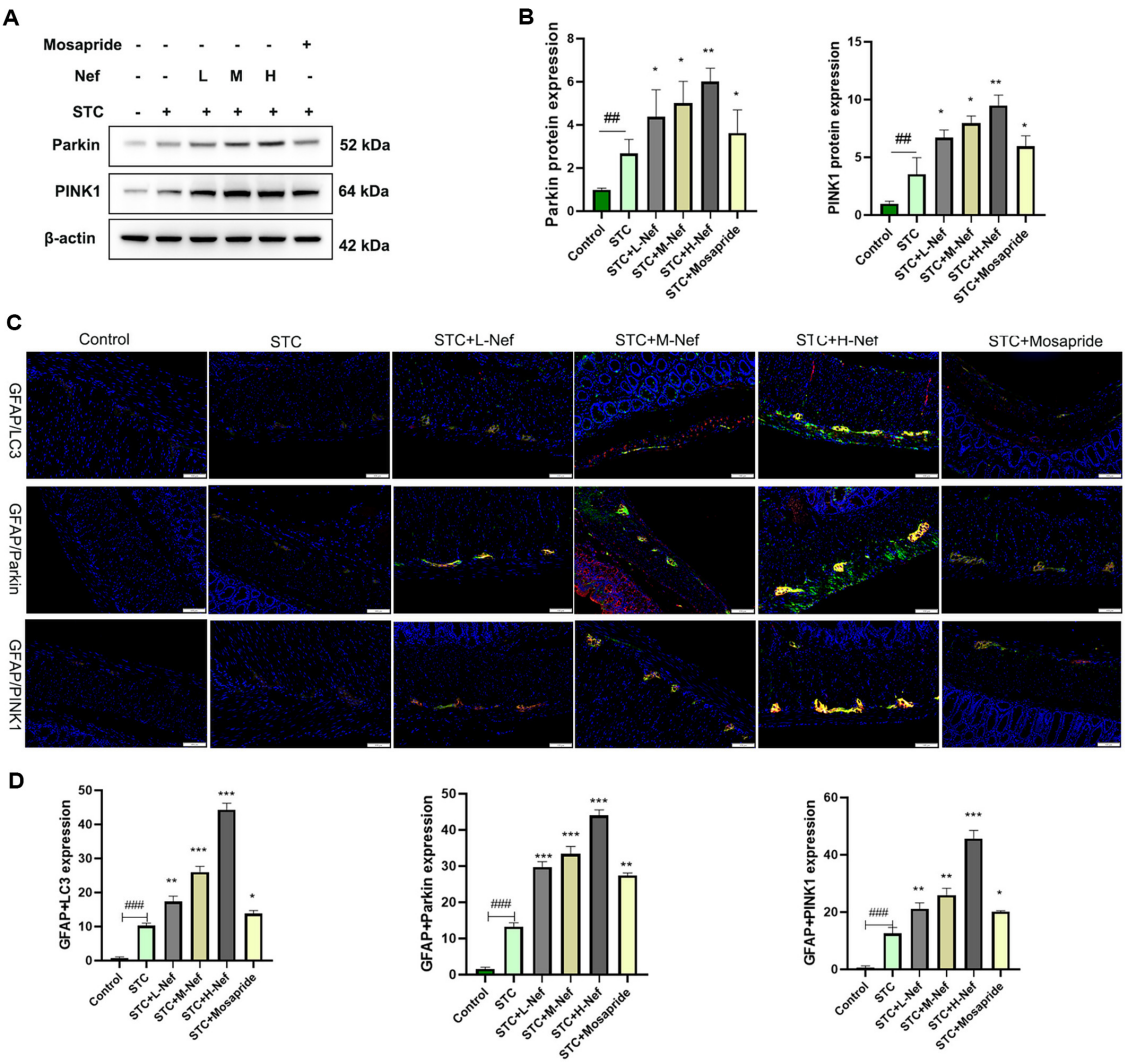
Effect of Nef on mitochondrial autophagy in EGCs in STC rats. (**A-B**) PINK1 and Parkin proteins were quantified by WB. (**C-D**) IF was performed to label GFAP/LC3, GFAP/Parkin, and GFAP/PINK1. Compared with the control group, ##*p* < 0.01, ###*p* < 0.001; Compared with the STC group, *p < 0.05, **p < 0.01, ***p < 0.001.

In addition, the protein expression of PINK1 and Parkin was obtained by western blotting (Fig. 4A). Fig. 4B shows that Nef could increase PINK1 and Parkin expression. GFAP was used to mark EGCs. Fig. 4C and 4D confirmed the activation of the PINK1/Parkin signaling in the STC+H-Nef group.

### Nef Targets PINK1 to Protect H_2_O_2_-Stimulated EGCs from Oxidative Stress

**Fig. 5:**
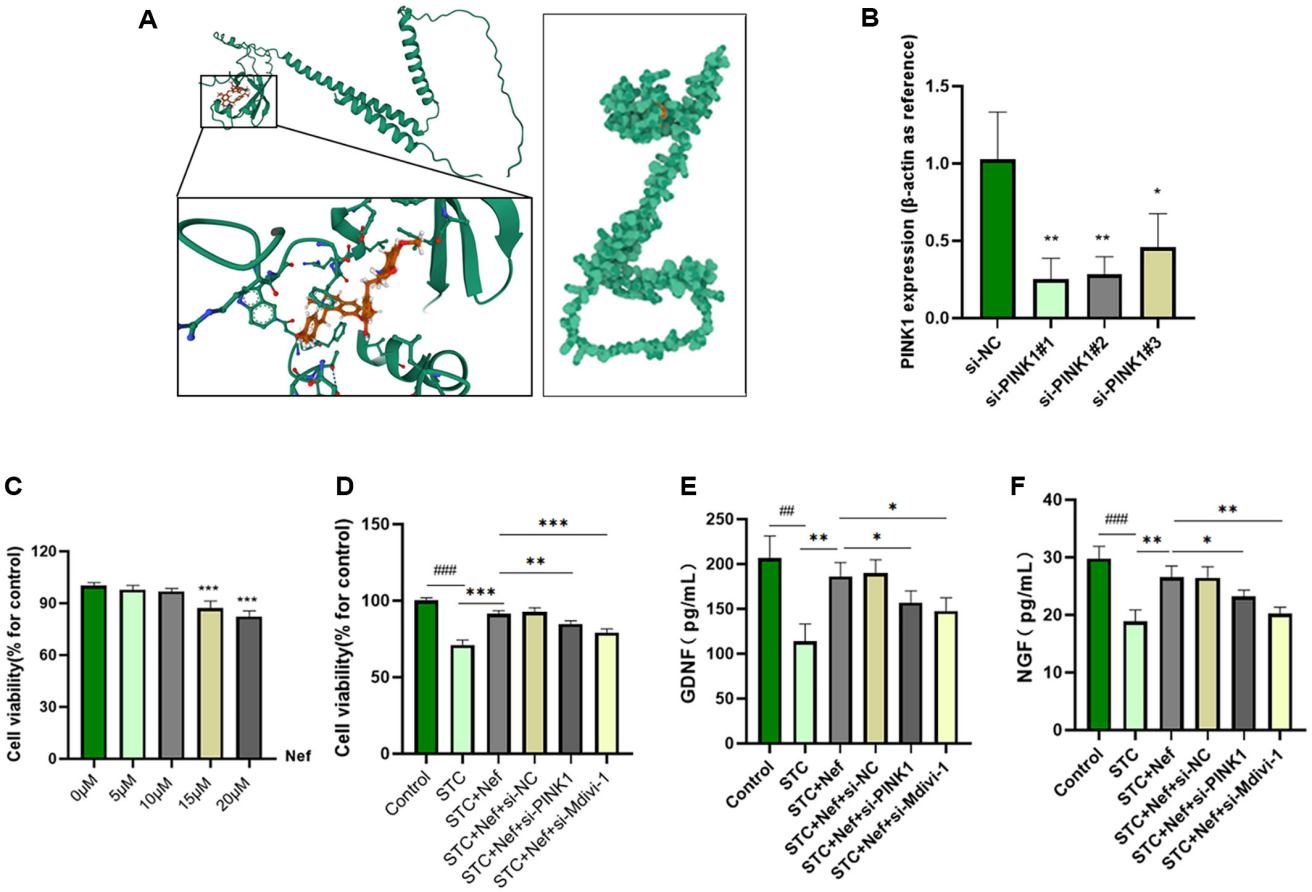
Nef targets PINK1 to protect H_2_O_2_-stimulated EGCs. (**A**) Molecular docking of PINK1 and Nef. (**B**) si-PINK1#1, si-PINK1#2, and si-PINK1#3 were transfected in EGCs, and qPCR was used to quantify PINK1 expression. (**C**) CCK-8 assay was conducted to measure cell viability under 0, 5, 10 15, and 20 μM Nef. (**D**) CCK-8 assay was conducted to measure cell viability under Nef and si-PINK treatment. ELISA was performed to detect GDNF (**E**) and NGF (**F**) levels. Compared with the control group, ##*p* < 0.01, ###*p* < 0.001; Compared with the STC+Nef group, *p < 0.05, **p < 0.01, ***p < 0.001.

Molecular docking was performed to evaluate the affinity of Nef for PINK1 (Fig. 5A). Results revealed that Nef can bind to PINK1, the most robust bond-binding energy formed was -6.929 kcal/mol, and the π-bond formed with phenylalanine (PHE 151) at position 151 of protein. To explore whether Nef targets PINK1 to protect EGCs from oxidative stress, the PINK1 was silenced by siRNA and co-treated with Nef in EGCs. si-PINK1#1, si-PINK1#2, and si-PINK1#3 were transfected in EGCs, and Fig. 5B demonstrated that si-PINK1#1 has the highest PINK1 expression knockdown efficacy. Next, 0, 5, 10, 15, and 20 μM Nef was used to treat EGCs and the cell viability was determined by CCK-8 (Fig. 5C). The results indicated that 15 and 20 μM Nef could damage EGCs. Thus, 10 μM Nef was applied for the following experiments. As shown in Fig. 5D, Nef could rescue los-induced EGCs but si-PINK1 transfection and Mdivi-1 (mitophagy inhibitor) treatment reversed it. Moreover, the GDNF (Fig. 5E) and NGF (Fig. 5F) levels are consistent with the cell viability trend when EGCs were treated by Nef, si-PINK1, or Mdivi-1.

**Fig. 6:**
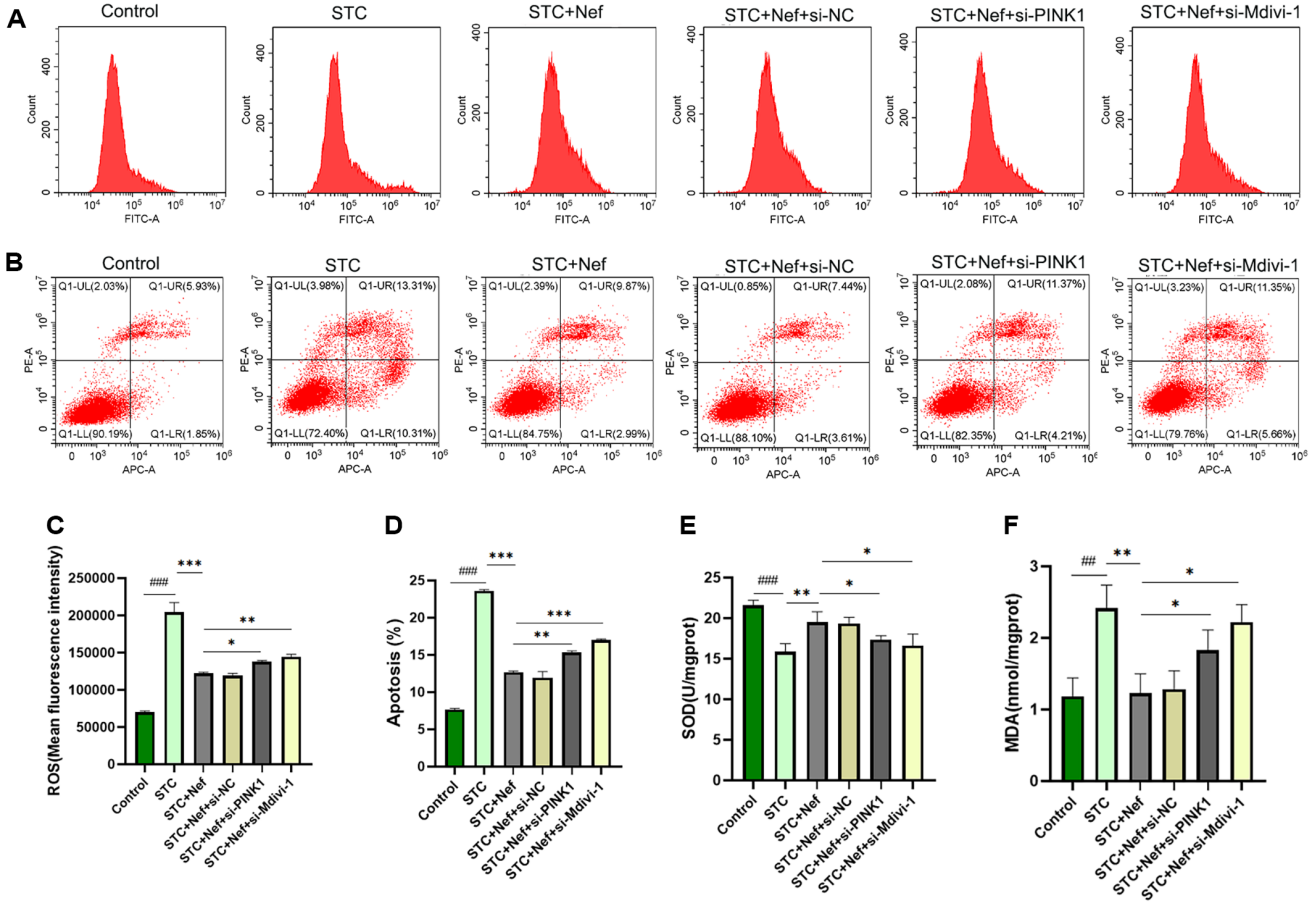
Nef targets PINK1 to inhibit oxidative stress and apoptosis in H_2_O_2_-induced EGCs. (**A, C**) FCM was used to measure ROS. (**B, D**) FCM was used to measure EGCs apoptosis. ELISA was performed to detect SOD (**E**) and MDA (**F**) levels in EGCs. Compared with the control group, ##*p* < 0.01, ###*p* < 0.001; Compared with the STC+Nef group, *p < 0.05, **p < 0.01, ***p < 0.001.

Furthermore, the ROS (Fig. 6A and 6C) and apoptosis (Fig. 6B and 6D) levels of EGCs were explored by FCM, and ELISA detected the SOD (Fig. 6E) and MDA (Fig. 6F) levels. H_2_O_2_-stimulated EGCs had increased ROS, apoptosis, and MDA levels. Compared with the STC group, the STC+Nef group revealed lower levels of ROS, apoptosis, and MDA. However, the si-PINK1 transfection and Mdivi-1 treatment caused an increase in these indices in comparison to the STC+Nef group. Moreover, the SOD expression in each group expressed the opposite trend to ROS, apoptosis, and MDA. These findings suggest that Nef may target PINK1 to protect H_2_O_2_-stimulated EGCs from oxidative stress, and the mechanism may link to mitophagy.

### Nef Protects EGCs by Inducing PINK1/Parkin-Mediated Mitochondrial Autophagy

**Fig. 7:**
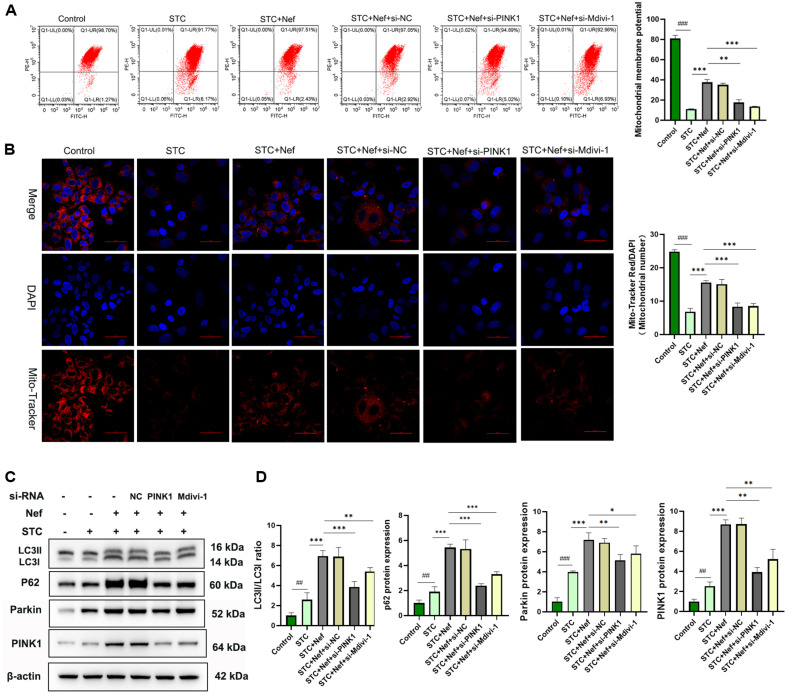
Nef protects EGCs by inducing the Pink1/Parkin-Mediated mitophagy. (**A**) FCM was used to measure mitochondrial membrane potential (MMP). (**B**) Immunofluorescence Mito-Tracker Red/DAPI staining to observe the number of mitochondria. (**C-D**) PINK1, Parkin, and LC3B proteins were quantified by WB. Compared with the control group, ##*p* < 0.01, ###*p* < 0.001; Compared with the STC+Nef group, *p < 0.05, **p < 0.01, ***p < 0.001.

The previous study inferred that Nef may protect H_2_O_2_-stimulated EGCs by promoting mitophagy. It is necessary to examine the mitochondrial injury. As displayed in Fig. 7A, MMP was destroyed by H_2_O_2_ stimulation, but alleviated by Nef treatment. Moreover, PINK1 silencing and Mdivi-1 treatment downregulated the MMP of EGCs in the STC+ Nef group. Mito-Tracker Red/DAPI staining was used to observe mitochondria (Fig. 7B). Results indicated that the number of mitochondria is consistent with the MMP trend. In addition, we detected p62, PINK1, Parkin, and LC3 protein expression using a western blot assay (Fig. 7C). Fig. 7D shows that Nef aggravated H_2_O_2_-stimulated upregulation of the LC3II/LC3I ratio and protein expression of p62, PINK1, and Parkin, but this was reversed by PINK1 silencing and Mdivi-1 treatment.

## Discussion

To explore whether Nef can alleviate the symptoms of constipation in STC rats, the body weight, fecal excretion, fecal water content (%), and intestinal propulsion rate (%) were recorded. Results revealed that Nef can upregulate these indices. In addition, Nef treatment significantly reduced the inflammatory cell infiltration and fibrous tissue proliferation of the colon in STC rats. These findings are consistent with previous studies. Previously, it has been shown that colonic fibrosis and inflammation are the key pathologies in STC model rats [16]. Y. Xia *et al*. reported that Nef can inhibit fibrosis endometriosis by mediating the TGF-β/ERK signaling pathway [17]. Furthermore, another study has demonstrated that Nef can activate 5-HT1A receptors, while 5-HT is essential for maintaining intestinal peristaltic function [18]. It suggested that Nef may be a potential drug for STC. However, the underlying molecular mechanism is still unclear.

To further determine the effects of Nef on STC rats, the ROS, SOD, and MDA levels of colon tissue were quantified. Nef was demonstrated to alleviate the Lop-induced oxidative stress to protect colon health. In this study, we found that the glial cell-line derived neurotrophic factor (GDNF) and NGF expression were upregulated by Nef in a dose-dependent manner. It is essential for maintaining the function of the enteric nervous system (ENS) that GDNF and NGF promote neuronal growth and protect neurons from oxidative stress [19, 20]. EGCs are one of the most important sources of GDNF and NGF, which have been proven to affect the barrier properties of the gut [21]. Thus, although it was speculated that Nef may improve STC by protecting EGCs, further evidence is required for substantiation.

It appears that oxidative stress causes mitochondrial damage due to ROS production. This type of damage requires the clearance of damaged mitochondria through mitophagy. In this study, the TEM images showed that STC induced mitochondria injury and Nef alleviated it, suggesting the Nef effects on STC may be related to mitophagy. Mitophagy is a critical cellular mechanism by which damaged mitochondria are selectively removed through the autophagy pathway. This process plays a key role in maintaining mitochondrial quality control and cellular homeostasis, thereby protecting cells from the adverse effects of mitochondrial dysfunction [22]. Thus, the mitophagy level increases indicate Nef protection for EGCs.

Pieces of evidence have demonstrated that the PINK1/Parkin signaling regulates mitophagy in multiple diseases, such as cardiomyopathy [23], Parkinson's disease[24], and kidney injury [25]. Specifically, when mitochondrial function is normal, PINK1 (a serine/threonine kinase) is transported to the inner mitochondrial membrane (IMM) and degraded. However, when the MMP is lost, PINK1 cannot be transported to IMM and accumulates on the outer mitochondrial membrane (OMM). This accumulation leads to the phosphorylation of downstream Parkin (an E3 ubiquitin ligase). Activated Parkin then ubiquitinates proteins on the OMM, which further bind to LC3 through autophagy receptors, inducing the formation of autophagosomes. Ultimately, the autophagosome encloses and degrades the damaged mitochondria [26, 27]. p62 contains an LC3-interacting region (LIR) through which it binds directly to LC3-II. The molecular forms of LC3 include LC3-I and LC3-II. During the process of mitophagy, LC3-I, as the precursor of LC3-II, is recruited to the vicinity of the expanding autophagosome membrane. Subsequently, it is converted into LC3-II through a series of enzymatic reactions. LC3-II plays a crucial role in maintaining the membrane stability of the substructures, facilitating their eventual fusion with lysosomes [28]. Therefore, LC3, PINK1, and Parkin must be detected to verify whether Nef alleviates STC by protecting EGCs. In animal experiments, the western blot results demonstrated that Nef promotes mitophagy via activating the PINK1/Parkin signaling pathway. Furthermore, a type III intermediate filament protein (GFAP) highly expressed in mature and activated EGCs was chosen as a marker for EGCs [29]. The IF double labeling demonstrated that the Nef could promote the PINK1/Parkin signaling pathway activation in EGCs of the colon.

In addition, molecular docking confirmed the binding energy and interaction between Nef and PINK1. Considering the PINK1 role in mitophagy, si-RNA was transfected into EGCs to knock down PINK1 expression, and the qPCR assay verified the PINK1 silencing efficiency. Mitochondrial division inhibitor 1 (Mdivi-1), a quinazolinone derivative, was selected as the mitophagy inhibitor in numerous studies [30, 31]. In the *in vitro* studies, it was employed to compare with si-PINK1 effects in the STC+Nef group. Results demonstrated that PINK1 silencing could reverse the Nef-induced upregulation of the GDNF and NGF expression, oxidative stress and apoptosis inhibition, and mitophagy promotion. Therefore, Nef can ameliorate STC rats by inducing the PINK1/Parkin signaling-mediated mitophagy in EGCs. In conclusion, this study provides a novel method to treat STC and theoretical guidance for the clinical application of Nef.

## Figures and Tables

**Table 1:** Nef improves loperamide hydrochloride-induced STC in SD rats.

	Control	STC	STC+L-Nef	STC+M-Nef	STC+H-Nef	STC+Mosapride
Weight	258.23 ± 4.49	259.52 ± 2.09	257.90 ± 6.69	259.08 ± 5.30	257.17 ± 6.66	257.57 ± 3.32
	407.58 ± 13.6***	350.55 ± 6.23	364.38 ± 6.90*	382.50 ± 5.17**	393.42 ± 18.6**	327.50 ± 5.27*
The amount of defecation (capsules)	35.17 ± 3.25***	11.17 ± 1.60	16.17 ± 3.06*	23.67 ± 3.01***	26.67 ± 1.86***	22.17 ± 1.72***
Fecal water content (%)	57 ± 2.21***	32 ± 1.63	35 ± 2.71*	44 ± 2.66***	46 ± 2.48***	40 ± 2.74***

Compared with the STC group, *p < 0.05, **p < 0.01, ***p < 0.001.
